# Bacterial endophytes from wild maize suppress *Fusarium graminearum* in modern maize and inhibit mycotoxin accumulation

**DOI:** 10.3389/fpls.2015.00805

**Published:** 2015-10-06

**Authors:** Walaa K. Mousa, Charles R. Shearer, Victor Limay-Rios, Ting Zhou, Manish N. Raizada

**Affiliations:** ^1^Department of Plant Agriculture, University of GuelphGuelph, ON, Canada; ^2^Department of Pharmacognosy, Mansoura UniversityMansoura, Egypt; ^3^Department of Plant Agriculture, University of GuelphRidgetown, ON, Canada; ^4^Guelph Food Research Centre, Agriculture and Agri-Food CanadaGuelph, ON, Canada

**Keywords:** *Fusarium graminearum*, Gibberella ear rot, maize, *Zea diploperennis*, parviglumis, endophyte, *Paenibacillus*, deoxynivalenol

## Abstract

Wild maize (teosinte) has been reported to be less susceptible to pests than their modern maize (corn) relatives. Endophytes, defined as microbes that inhabit plants without causing disease, are known for their ability to antagonize plant pests and pathogens. We hypothesized that the wild relatives of modern maize may host endophytes that combat pathogens. *Fusarium graminearum* is the fungus that causes Gibberella Ear Rot (GER) in modern maize and produces the mycotoxin, deoxynivalenol (DON). In this study, 215 bacterial endophytes, previously isolated from diverse maize genotypes including wild teosintes, traditional landraces and modern varieties, were tested for their ability to antagonize *F. graminearum in vitro*. Candidate endophytes were then tested for their ability to suppress GER in modern maize in independent greenhouse trials. The results revealed that three candidate endophytes derived from wild teosintes were most potent in suppressing *F. graminearum in vitro* and GER in a modern maize hybrid. These wild teosinte endophytes could suppress a broad spectrum of fungal pathogens of modern crops *in vitro*. The teosinte endophytes also suppressed DON mycotoxin during storage to below acceptable safety threshold levels. A fourth, less robust anti-fungal strain was isolated from a modern maize hybrid. Three of the anti-fungal endophytes were predicted to be *Paenibacillus polymyxa*, along with one strain of *Citrobacter*. Microscopy studies suggested a fungicidal mode of action by all four strains. Molecular and biochemical studies showed that the *P. polymyxa* strains produced the previously characterized anti-*Fusarium* compound, fusaricidin. Our results suggest that the wild relatives of modern crops may serve as a valuable reservoir for endophytes in the ongoing fight against serious threats to modern agriculture. We discuss the possible impact of crop evolution and domestication on endophytes in the context of plant defense.

## Introduction

Modern maize, belonging to the genus *Zea*, was domesticated in southern Mexico 9000 years ago from wild, annual tropical grasses called teosintes, with the primary ancestor being Parviglumis (*Zea mays* ssp. parviglumis) which survives today in the wild (Matsuoka et al., 2002). There are additional species of teosintes that continue to grow in the wild in Mexico and Central America including the perennial *Zea diploperennis* (Iltis and Doebley, 1980). Following its domestication into an edible crop (*Z. mays* ssp. mays), maize was bred and spread by indigenous farmers throughout the Americas to give rise to diverse traditional landraces (Matsuoka et al., 2002). In the Twentieth Century, scientists created improved, commercial inbreds and hybrids such as the temperate hybrid, Pioneer 3751 (Smith et al., 2004). Wild maize has been reported to be more resistant to pests than their modern counterparts, perhaps due to loss of defense alleles and/or loss of the protective casing (fruitcase) enclosing the grains in modern varieties, as a result of breeding and domestication (Wang et al., 2005; Lange et al., 2014).

Though the increased disease susceptibility of modern maize has been attributed to changes in the plant genome, there may be additional explanations. Endophytes are microbes that inhabit the internal tissues of plants, including seeds, without causing disease symptoms (Wilson, 1995; Johnston-Monje and Raizada, 2011; White and Bacon, 2012; Mousa and Raizada, 2013). Some endophytes have been shown to help their host plants to combat pathogens (Mousa and Raizada, 2013, 2015). During maize evolution, domestication, breeding and migration, some endophytes were lost (Johnston-Monje and Raizada, 2011; Johnston-Monje et al., 2014), and it is also possible that endophytic genomes may have been modified—phenomena that might contribute to the increased disease susceptibility of modern maize.

Modern maize is susceptible to various pathogens including *Fusarium graminearum*, the fungus that causes Gibberella Ear Rot (GER). GER is a serious global disease particularly in Europe, the United States and Canada (van der Lee et al., 2014). In grain, *F. graminearum* produces deoxynivalenol (DON), a mycotoxin that inhibits DNA and protein synthesis, resulting in various toxicity effects in both humans and animals (Munkvold, 2003b; Voss, 2010; Hassan et al., 2015). In a 3 year survey, 59% of maize samples tested from around the world were found to be contaminated with DON (Rodrigues and Naehrer, 2012).

Although the current disease management strategies to combat *F. graminearum* rely on breeding for resistance genotypes, optimizing cultural practices or use of fungicides, these strategies have achieved low to moderate success (Munkvold, 2003a; Edwards, 2004; Reid et al., 2009; Wegulo et al., 2011). A promising alternative strategy to manage *Fusarium* outbreaks and reduce mycotoxin contamination may be through the use of biological antagonists (Eilenberg, 2006; Bacon and Hinton, 2011; Chulze et al., 2014). We have recently reported that wild, traditional and modern maize possess endophytes that combat pathogens including *F. graminearum in vitro* (Johnston-Monje and Raizada, 2011; Johnston-Monje et al., 2014). Other studies have identified other biological control agents that combat *F. graminearum* including *Bacillus* and *Pseudomonas* spp. (Moussa et al., 2013; Shi et al., 2014). However, most of this research is preliminary, and effective commercial biological control is not currently available.

Here, we tested the hypothesis that the wild relatives of maize may possess endophytes that help their hosts to naturally combat *F. graminearum*. A library of bacterial endophytes, previously isolated from diverse maize genotypes including wild, traditional and modern varieties (Johnston-Monje and Raizada, 2011; Johnston-Monje et al., 2014), were screened for their ability to inhibit the growth of *F. graminearum in vitro* and suppress GER *in planta*.

## Materials and methods

### Source of bacterial endophytes

A library of 215 bacterial endophytes was previously isolated in our lab to study the diversity of maize microbial endophytes (Johnston-Monje and Raizada, 2011). The endophytes were isolated from 14 diverse *Zea* genotypes, including wild teosintes (*Zea mays* ssp. *parviglumis, Zea mays* ssp. *mexicana, Zea diploperennis, Zea nicaraguensis*), ancient and traditional Mexican landraces of modern maize (*Zea mays* ssp. *mays:* Gaspe yellow Flint, Cristalino de Chihuahua, Chapalote, Mixteco, Bolita, Jala, Nal-Tel, Tuxpeno) and modern maize varieties (*Zea mays* ssp. *mays*: Pioneer 3751 and B73). The endophytes analyzed from wild, traditional and modern genotypes represented 46, 33, and 21% of the library, respectively, with approximately half the library isolated from non-wild, post-domesticated maize (54%) (Table S3).

### Antifungal screening

Overnight cultures of each endophyte were used to screen endophytic bacteria for *in vitro* inhibition of growth of *F. graminearum* (obtained from Agriculture and Agrifood Canada, Guelph, ON) using the dual culture method. Each bacterial endophyte was cultured in liquid broth (LB, Luria-Bertani, composed of 10 g NaCl, 5 g yeast extract, 10 g tryptone, per liter), grown for 1–3 days at 37°C with shaking at 225 rpm then centrifugation for 5 min, followed by resuspension in PBS buffer to an OD_600_ of 0.4–0.6 (Spectromax, serial # MN03135, USA). *F. graminearum* was grown for 48 h (25°C, 100 rpm) in liquid potato dextrose broth media (Catalog # P6685, Sigma Aldrich, USA), then mycelia was added to melted, cooled PDA media (1 ml of fungus into 100 ml of media), mixed and poured into Petri dishes (100 × 15 mm), then allowed to re-solidify. Wells (11 mm diameter) were created in this pathogen-embedded agar by puncturing with sterile glass tubes, into which the endophyte cultures were applied (200 μl per well). The agar plates were incubated at 30°C for 48 h in darkness. The radius of each zone of inhibition was measured (mm). The commercial broad-spectrum fungicides, Amphotericin B (Catalog #A2942, Sigma Aldrich, USA) and Nystatin (Catalog #N6261, Sigma Aldrich, USA), were used as positive controls at concentrations of 5 and 10 μg/ml, respectively. LB was used as a negative control. Each endophyte was screened in three independent replicates.

### Anti-fungal target spectrum of the candidate endophytes

Endophytes that tested positive for activity against *F. graminearum* were re-screened for activity against a diversity of other fungal species including crop pathogens (from the Agriculture and Agrifood Canada Fungal Type Collection, Guelph, ON, Canada) using the dual culture method (described above) to characterize the activity spectrum of each endophyte. The crop fungi tested included: *Alternaria alternata, Alternaria arborescens, Aspergillus flavus, Aspergillus niger, Bionectria ochroleuca, Davidiella* (*Cladosporium*) *tassiana, Diplodia pinea, Diplodia seriata, Epicoccum nigrum, Fusarium lateritium, Fusarium sporotrichioides, Fusarium avenaceum* (*Gibberella avenacea*, two isolates), *Nigrospora oryzae, Nigrospora sphaerica, Paraconiothyrium brasiliense, Penicillium expansum, Penicillium afellutanum, Penicillium olsonii, Rosellinia corticium, Torrubiella confragosa, Trichoderma hamatum* and *Trichoderma longibrachiatum*.

### Molecular identification of candidate endophytic bacteria using 16S rDNA and 23S rDNA

For taxonomic identification of candidate endophytic bacteria, a standard PCR protocol was used (Johnston-Monje and Raizada, 2011). Bacterial genomic DNA was extracted (GenElute Bacterial Genomic DNA kit, NA2110-1KT, Sigma) and quantified using a Nanodrop machine (Thermo Scientific, USA). The extracted DNA was used to amplify 16S rDNA and 23S rDNA using PCR.

#### For 16S rDNA amplification

A PCR master mix (20 μl) was made as follows: 50 ng DNA were added (2.5 ng/μl was the final DNA concentration in the PCR reaction), 2.5 μl Standard Taq Buffer (10 ×) (New England Biolabs), 0.5 μl of 25 mM dNTP mix, 1 μl of 10 mM 1492r primer with sequence GGTTACCTTGTTACGACTT, 1 μl of 10 mM 799f primer with sequence AACMGGATTAGATACCCKG (M and K refer to degenerate nucleotides, where M is A or C and K is G or T), 0.25 μl of 50 mM MgCl_2_, 0.25 μl of Standard Taq (10 U/μl, New England Biolabs), and double distilled water up to 20 μl total.

#### For 23S rDNA amplification

A PCR master mix (20 μl) was made as described above using 1 μl of 10 mM 23S 6F primer with sequence 5′-GCGATTTCYGAAYGGGGRAACCC and 1 μl of 10 mM 23S R primer with sequence 5′- TTCGCCTTTCCCTCACGGTACT (where Y is C or T and R is A or G) (Anthony et al., 2000).

For both 16S rDNA and 23S rDNA, the PCR amplification conditions were: 96°C for 3 min, followed by 35 amplification cycles (94°C for 30 s, 48°C for 30 s, 72°C for 90 s), and a final extension at 72°C for 7 min, using a PTC200 DNA Thermal Cycler (MJ Scientific, USA). Finally, the PCR products were separated on 1.5% agarose gels at ≤ 5 V/cm, then the bands were visualized under UV light; 700 and 400 bp bands were excised for 16S rDNA and 23S rDNA, respectively and eluted from the gels (Illustra GFX 96 PCR Purification kit, GE Healthcare, USA). The purified DNA was sequenced at the Genomic Facility Laboratory at the University of Guelph. For 16S, primers 1492r and 799f were used for sequencing, while for 23S, primer 23S 6F was used. Bacterial strains were identified based on 16S rDNA and 23S rDNA sequence comparisons using BLAST searches to GenBank. To assist with taxonomic identification, 16S rDNA sequences for the three *Paenibacillus* sp. were used to generate a phylogenetic tree using Phylogeny.fr (Dereeper et al., 2008, 2010).

### Scanning electron microscope (SEM) imaging of endophytes

Scanning electron microscopy imaging was conducted to visualize the external appearance of candidate bacteria following a standard protocol (Hayat, 1989). Bacterial cultures were plated on LB plates, incubated for 24 h then suspended and washed in phosphate buffer (pH 7). A drop of the suspension was placed on a carbon disc and left to dry for 1 h. The dried bacteria was washed with phosphate buffer then fixed by adding 2% glutaraldehyde for 1 h. The fixed bacteria was then treated with 1% osmium tetroxide for 30 min, then gradually dehydrated using an ethanol series (50, 70, 80, 90, and 100%) followed by critical point drying. The dried bacterial films were coated with gold and examined by SEM (Hitachi S-570 SEM, Hitachi High Technologies, Tokyo, Japan) at the Imaging Facility, Department of Food Science, University of Guelph.

### *In vitro* interaction between each endophyte and *F. graminearum*

The *in vitro* interaction between *F. graminearum* and each bacterial endophyte was studied microscopically. Each microscope slide was coated with a thin layer of PDA, then 50 μl of bacterial endophyte culture (grown overnight in LB incubated at 37°C, 250 rpm) was applied adjacent to 50 μl of *F. graminearum* mycelia (grown for 24–48 h in potato dextrose broth at 25°C, 100 rpm). Each slide was incubated at 25°C for 24 h then stained with the vitality stain, neutral red (Sigma Aldrich, Catalog #57993) or Evans blue (Sigma Aldrich, Catalog # E2129) by placing 100 μl of stain on the slide, followed by a 3–5 min incubation at room temperature, then washing 3–4 times with deionized water. A commercial biological control agent with a fungicidal mode of action (*Bacillus subtilis* QST713, Bayer CropScience, Batch #00129001) was used as a positive control (100 mg/10 ml). Pictures were taken using light microscopes (MZ8, Leica, Wetzlar, Germany for neutral red staining; and a BX51 microscope, Olympus, Tokyo, Japan for Evans blue staining). There were 3–4 replicates for each slide.

### PCR based approach to detect the fusaricidin biosynthetic gene in the *paenibacillus* endophyte strains

In order to detect the presence of a candidate fusaricidin synthetase gene (*fusA*) in the *Paenibacillus* endophyte strains, two oligonucleotides (FusAF and FusAR) were designed based on the *fusA* sequence (GenBank accession #EU184010) using Primer3 software. For *fusA* amplification, a PCR master mix (20 μl) was made as follows: 50 ng DNA were added (2.5 ng/μl was the final DNA concentration in the PCR reaction), 50 ng DNA, 2.5 μl Standard Taq Buffer (10 ×) (New England Biolabs), 0.5 μl of 25 mM dNTP mix, 0.25 μl of 50 mM MgCl_2_, 0.25 μl of Standard Taq (10 U/μl, New England Biolabs), 1 μl of primer FusAF with sequence 5′- AGGCAAGCTTTGACTTGGAA −3′ and 1 μl of primer FusAR with sequence 5′- CGCTTGCTCAGACCATACAA −3′ and double distilled water up to 20 μl total. The PCR amplification conditions were: 96°C for 3 min, followed by 35 amplification cycles (94°C for 30 s, 48°C for 30 s, 72°C for 90 s), and a final extension at 72°C for 7 min, using a PTC200 DNA Thermal Cycler (MJ Scientific, USA). The PCR products were separated on 1.5% agarose gels at ≤ 5 V/cm, then the bands were visualized under UV light; bands were excised and eluted from the gels (Illustra GFX 96 PCR Purification kit, GE Healthcare, USA). The purified DNA was sequenced at the Genomic Facility Laboratory at the University of Guelph. The corresponding gene was identified based on best BLAST matches to Genbank.

### Biochemical detection of fusaricidins in endophyte culture filtrates using LC-MS

To detect the presence of fusaricidins biochemically in *Paenibacillus* spp., endophytes were grown for 48 h on Katznelson and Lochhead liquid medium (Paulus and Gray, 1964), harvested by freeze drying, then the lyophilized powder from each strain was extracted by methanol. The methanolic extracts were run on a Luna C18 column with a gradient of 0.1% formic acid and 0.1% formic in acetonitrile. Peaks were analyzed by mass spectroscopy (Agilent 6340 Ion Trap), ESI, positive ion mode. LC-Mass analysis was conducted at the Mass Spectroscopy Facility, McMaster University, Ontario, Canada. The m/z ratios were compared to the published literature (Kajimura and Kaneda, 1996, 1997; Beatty and Jensen, 2002).

### GFP-tagging for ecological tracking *in planta*

In order to test the ability of candidate anti-*Fusarium* endophytes to colonize a modern maize hybrid, the endophytes were subjected to tagging with green fluorescent protein (GFP) followed by *in planta* visualization using confocal scanning microscopy.

#### To prepare competent cells

One liter of LB broth was inoculated with 10 ml bacterial culture grown overnight (37°C at 250 rpm) until early log phase (OD_600_ = 0.4–0.6). The cells were harvested by chilling for 15 min on ice and centrifuged at 4000 × *g* for 15 min at 4°C. The pellets were then re-suspended in cold water and centrifuged two times. Finally, the pellets were re-suspended in cold 10% glycerol, centrifuged and re-suspended in 3 mL of 10% glycerol from which 40 μl aliquots were made and frozen at –80°C.

#### GFP plasmid transformation

A wide-host promoter plasmid, pDSK-GFPuv (Wang et al., 2007) was used to transform *E.coli* DH5α (Catalog #EC6P095H, Epicenter, Madison, USA), which was then stored at −80°C. The plasmid was extracted from *E.coli* using a standard protocol (Catalog # 732-6100, BioRad Inc., USA) then introduced into bacterial cells by electroporation (Calvin and Hanawalt, 1988). Suspensions of 40 μl cold competent cells were mixed with 1 μl of plasmid DNA (240 ng/μl) then electroporated at 1.6 KV for 1 s using a Bio-Rad Gene Pulser 200/2.0 (Bio-Rad Hercules, USA). After electroporation, cells were incubated for 1 h in 1 ml of LB at 37°C with shaking at 250 rpm. Transformed cells were plated on LB agar containing Kanamycin (35 μg/μl) and incubated for 24 h at 37°C, then the plate was examined for fluorescent colonies (Illumatool, #LR 92240, Lightools Research, USA).

#### To visualize GFP-tagged endophyte cells inside maize tissues

Modern maize seeds (Ontario maize hybrid P35F40, see below) were surface sterilized, coated with GFP-tagged endophyte (see below for details), and planted on wet paper towels. One-week-old seedlings were stained with propidium iodide (1 mg/ml) (Sigma Aldrich, Catalog #P4170) then washed with deionized water. The seedlings were screened by a TCS SP2 confocal laser scanning microscope (Leica Microsystems, Mannheim, Germany) at the Imaging Facility, University of Guelph. The conditions for confocal microscopy were as follows: excitation at 488 nm with an Argon laser and at 543 nm with a green helium laser (emission ranges = 504–532 nm and 524–699 nm, respectively), pinhole [Au] = 1.0 airy, objective lens = 63 × oil immersion, and frame average = 3 times.

### Suppression of GER in greenhouse trials

The candidate endophytes that suppressed the growth of *F. graminearum in vitro* were tested for their ability to suppress GER in greenhouse trials (Crop Science Greenhouse Facility, University of Guelph):

#### Seed treatment

Seeds of a susceptible commercial maize hybrid (P35F40) were surface sterilized as follows: seeds were washed in 0.1% Triton X-100 detergent for 10 min with shaking; the detergent was decanted, 3% sodium hypochlorite was added for 10 min, followed by rinsing with autoclaved, distilled water, washing with 95% ethanol for 10 min; and finally the samples were washed 5–6 times with autoclaved, distilled water. Effective surface sterilization was ensured by inoculating the last wash on LB and PDA plates at 37 and 25°C, respectively; all washes showed no growth. The sterilized seeds were then coated with endophytic inoculants (on the day of planting). To prepare endophytic bacterial inoculants, bacteria were grown for 24 h at 37°C in liquid LB medium, centrifuged, washed and suspended in PBS buffer to an OD_600_ of 0.5. Thereafter, 500 μl of each bacterial suspension were mixed with 10 ml polyvinyl pyrrolidine (PVP, Catalog # 9003398, Sigma Aldrich, USA) as a seed-coating agent; then incubated with the seeds for 2 h on a horizontal shaker (Serial #980216M, National Labnet Company, USA). Seeds coated with the endophytes or buffer control were then germinated on wet paper towels and kept in the dark for 7 days; uniformly sized seedlings were transferred into pots containing Turface clay (Turface Athletics Inc., USA) in the greenhouse under the following growth conditions: (28°C/20°C, 16 h:8 h, ≥ 800 μmol m^−2^ s^−1^ at pot level, with high pressure sodium and metal halide lamps supplemented with GroLux bulbs) using drip irrigation with modified Hoagland's solution until maturity (Gaudin et al., 2011).

#### Pathogen introduction

Pathogen spores were prepared as follows: the liquid medium used for spore suspension was prepared with the following composition per liter in distilled water: 2 g KH_2_PO_4_, 2 g KNO_3_, 1 g MgSO_4_, 1 g KCl, 1 g dextrose, 20 mg/100 ml (of each) of minor elements (FeCl_2_, MnSO_4_, ZnSO_4_). Approximately 350 ml of the liquid medium was added to 2 L flasks and autoclaved at 121°C for 10 min. After cooling to room temperature, either 3–4 PDA plugs of *F. graminearum* isolate or 10 ml of liquid conidia suspension was added aseptically to each flask, which was incubated on a shaker table at room temperature under 12:12 h UV light: dark cycle for approximately 2 weeks. Using a haemocytometer, the solution was standardized to 20,000 spores/ml before being stored in the fridge or used in the greenhouse directly. One ml of *F. graminearum* spore suspension was applied first to silks beginning after their emergence.

#### Endophyte silk spray treatment

To ensure high titre of the endophytes, they were introduced a second time, by spraying 1 ml of each endophyte (OD_600_ of 0.5, grown in LB) simultaneously with the pathogen inoculant, and then again at 3 days after pathogen inoculation.

#### Control treatments

For the positive control group, seeds were coated only with PVP followed by prothioconazole fungicide spraying (PROLINE® 480 SC, Bayer Crop Science) at the post-silking stage prior to infection with the fungal pathogen. The negative control was seeds coated only with PVP, then sprayed at silking with 1 ml of *F. graminearum* spore suspension only.

#### Experimental design

There were 20 plants arranged in a randomized block design per each treatment group. The trial was repeated independently in the summers of 2012 and 2013. In the second trial, to increase disease severity, the humidity around the ears was artificially increased by placing plastic bags around the ears. The plants were grown until full maturity.

#### Disease assessments

At full maturity stage, ears were phenotyped visually for the percentage of apparent infection, scored as the length of diseased area from the ear tip (infection site) relative to the total length of each respective ear. The other phenotype measured was average grain yield per plant (g) at harvest. Results were analyzed and compared by Mann-Whitney *t*-tests (*P* < 0.05).

### Suppression of DON production

Maize kernels were ground for 40 s to a texture that would pass through a 20-mesh sieve using an M2 Stein mill (Fred Stein Lab, Inc. Atchinson, KS, USA). Ground samples (5 g) were diluted in distilled water at a ratio of 1:5 (w/v) and shaken vigorously for 3 min using a bench top reciprocal Eberbach shaker equipped with a flask carrier (Eberbach Corp, Ann Arbor, MI).

A 2 ml aliquot of the suspension fluid was transferred into a microcentrifuge tube and spun at 8000 rpm for 60 s. Sample aliquots were subsequently diluted in distilled water when appropriate. ELISA analysis was carried out with the EZ-ToxTM DON Test (Diagnostix Ltd., Mississauga, ON, Canada) following the manufacturer's protocol with a detection limit of 0.1 μg/g. There were three replicates for each treatment. Results were analyzed and compared by the Mann-Whitney *t*-test (*P* < 0.05).

### DON detoxification

To test for the ability of the candidate endophytes to directly detoxify DON into epi- DON, *in vitro* liquid chromatography coupled to a diode array detector (LC-DAD) was used. Endophytes were grown in 5 ml LB for 48 h at 250 rpm with 20-ppm deoxynivalenol (DON, Catalog #D0156, Sigma-Aldrich, USA). The cultures were diluted to 2 ppm DON with MiliQ water. Then, DON was extracted from the bacterial cultures using monoclonal antibody-based affinity chromatography (VICAM, DONtest™ HPLC, G1005) then subjected to HPLC analysis to detect DON and epi-DON compared to control buffers. The samples were run on a C18 column (250 × 4.6 mm, product # 00G-4396-E0, Phenomenex Inc., USA) with an isocratic water- acetonitrile (90:10) system. Peaks were detected by photodiode array spectrophotometer equipped within an Agilent 1200 Infinity Series HPLC (Agilent, USA). There were three replicates for each endophyte tested.

### Statistical analyses

All statistical analysis was performed using Prism Software version 5 (GraphPad Software, USA).

## Results

### Antifungal screening

**Figure 1:**
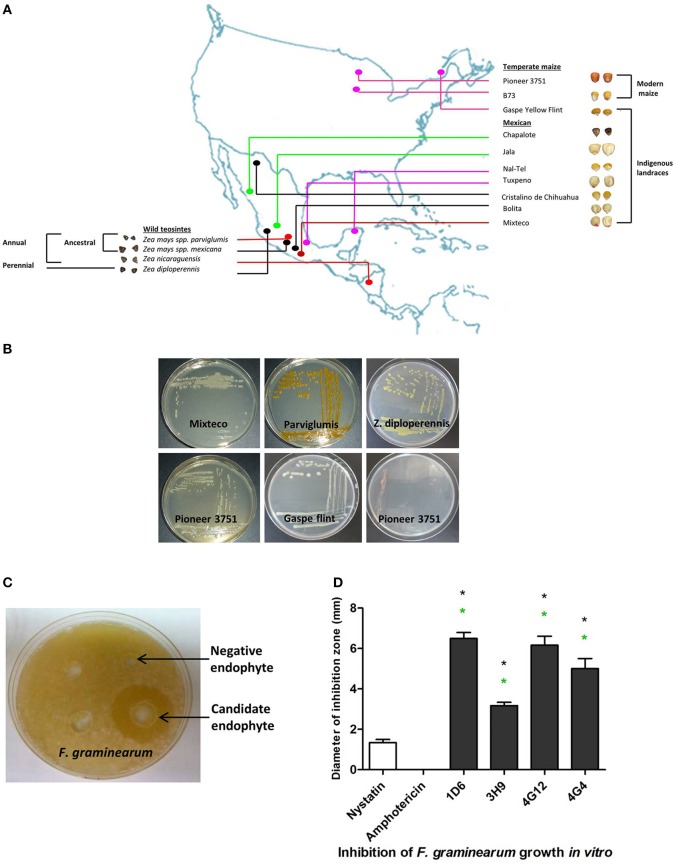
**Origin of endophytes used in this study and results of the *in vitro* anti-*Fusarium* screen. (A)** A map showing the origin of maize genotypes previously used to isolate the endophytic library. **(B)** Examples of endophytes from the library isolated from different *Zea* genotypes as indicated. **(C)** Example of an endophyte culture showing suppression of *F. graminearum* hyphae (white) using the dual culture method. **(D)** Quantification of the inhibitory effect of the endophytes or fungicide controls, amphotericin B and nystatin (at concentrations of 5 and 10 μg/ml, respectively), on the growth of *F. graminearum in vitro*. For these experiments, *n* = 3. The error bars indicate the standard error of the mean. The black asterisk indicates that the treatment means are significantly different from the fungicide Nystatin at *p* ≤ 0.05. The green asterisk indicates that the treatment means are significantly different from the fungicide Amphotericin at *p* ≤ 0.05.

**Figure 2:**
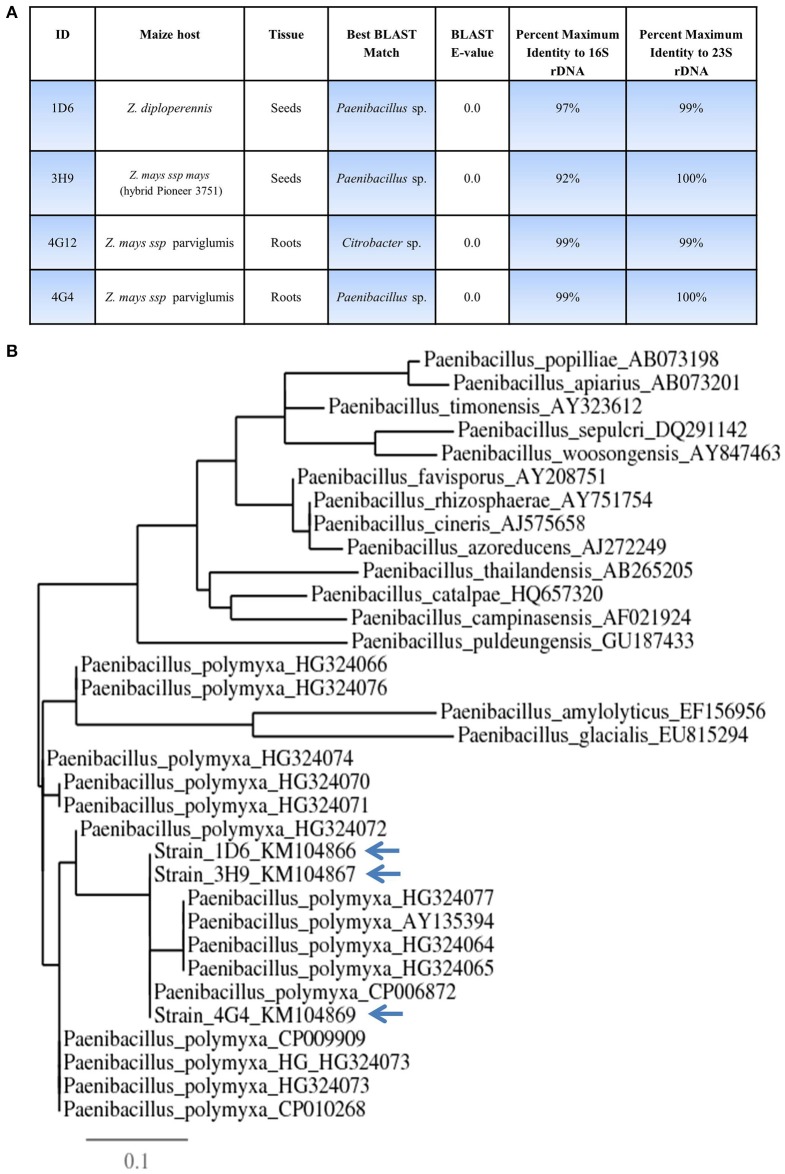
**Taxonomic characterization of candidate anti-*Fusarium* endophytes. (A)** Details of the taxonomic identification of the anti-*Fusarium* endophytes using 16S rDNA and 23S rDNA, and the tissue and host from which the endophytes were originally isolated. **(B)** 16S rDNA based phylogenetic tree of the three predicted *Paenibacillus* sp.

The dual culture method was used to screen 215 bacterial endophytes, previously isolated from diverse maize genotypes (Figures 1A,B), for their ability to suppress the growth of *F. graminearum*. Zones of inhibition of *F*. *graminearum* were measured after 24–48 h of co-incubation (Figures 1C,D). The results revealed that four bacterial endophytes could consistently inhibit the growth of *F. graminearum* (strains 1D6, 3H9, 4G12, and 4G4). Strain 1D6 resulted in the greatest growth inhibition, while 3H9 caused the least growth inhibition to *F. graminearum* (Figure 1D). Three of these endophytes were isolated from wild maize genotypes (teosintes): strain 1D6 from *Z. diploperennis* and strains 4G12 and 4G4 from Parviglumis, the direct ancestor of modern maize (Figures 1A,2A). The remaining candidate endophyte (strain 3H9) was isolated from a modern commercial variety (*Z. mays* ssp. *mays*, Pioneer 3751 hybrid).

### Anti-fungal target spectrum of the candidate endophytes

Using the dual culture method, endophytes that tested positive for activity against *F. graminearum*, were re-screened for activity against a collection of fungi including crop pathogens. Each candidate endophyte was screened in three independent replicates. Endophytes 1D6, 4G12, and 4G4 from the wild teosintes showed the highest spectrum of activity as they inhibited the growth of 20, 19, and 20 fungi, respectively, out of 20 tested fungi (other than *F. graminearum*). Endophyte 3H9 from modern maize showed a narrow activity spectrum as it inhibited the growth of only one fungus in addition to *F. graminearum* (Table 1).

**Table 1:** **Effect of the candidate bacterial endophytes on the growth of diverse crop fungal pathogens *in vitro***.

**Target fungal species**	**Mean diameter of inhibition zone with each endophyte (mm)**					
	**Nystatin (10 μg/ml)**	**Amphotericin (5 μg/ml)**	**1D6**	**3H9**	**4G12**	**4G4**
*Alternaria alternata*	0.0 ± 0.0	0.0 ± 0.0	3.0 ± 0.2^*^#	0.0 ± 0.0	4.5 ± 0.2^*^#	5.0 ± 0.0^*^#
*Alternaria arborescens*	0.0 ± 0.0	0.0 ± 0.0	5.5 ± 0.2^*^#	0.0 ± 0.0	5.5 ± 0.2^*^#	5.0 ± 0.2^*^#
*Aspergillus flavus*	2.0 ± 0.2	0.0 ± 0.0	5.5 ± 0.3^*^#	0.0 ± 0.0^*^	3.5 ± 0.2^*^#	4.0 ± 0.0^*^#
*Aspergillus niger*	0.0 ± 0.0	2.0 ± 0.0	6.5 ± 0.3^*^#	0.0 ± 0.0 #	5.0 ± 0.0^*^#	7.0 ± 1.0^*^#
*Bionectria ochroleuca*	2.0 ± 0.2	0.5 ± 0.2	5.5 ± 0.2^*^#	3.5 ± 0.3^*^#	6.0 ± 0.2^*^#	6.5 ± 0.2^*^#
*Davidiella tassiana*	1.5 ± 0.2	0.5 ± 0.3	5.0 ± 0.3^*^#	0.0 ± 0.0^*^#	4.5 ± 0.7^*^#	5.0 ± 0.0^*^#
*Diplodia pinea*	2.5 ± 0.2	3.0 ± 0.2	6.5 ± 0.3^*^#	0.0 ± 0.0^*^#	5.5 ± 0.2^*^#	6.0 ± 0.0^*^#
*Diplodia seriata*	3.0 ± 0.2	2.0 ± 0.2	3.0 ± 0.2#	0.0 ± 0.0^*^#	0.0 ± 0.0^*^#	1.5 ± 0.2^*^#
*Epicoccum nigrum*	0.0 ± 0.0	0.0 ± 0.0	1.5 ± 0.2^*^#	0.0 ± 0.0	4.0 ± 0.0^*^#	3.0 ± 0.2^*^#
*Fusarium avenaceum* (isolate 1)	2.5 ± 0.3	3.0 ± 0.6	7.0 ± 0.2^*^#	0.0 ± 0.0^*^#	4.5 ± 0.2^*^#	3.0 ± 0.2^*^
*Fusarium graminearum*	1.5 ± 1.6	0.0 ± 0.0	6.5 ± 0.3^*^#	3.0 ± 0.2^*^#	6.0 ± 0.4^*^#	5.0 ± 0.5^*^#
*Fusarium lateritium*	0.0 ± 0.0	1.0 ± 0.2	1.5 ± 0.3^*^#	0.0 ± 0.0 #	4.0 ± 0.5^*^#	5.5 ± 0.3^*^#
*Fusarium sporotrichioides*	1.0 ± 0.2	1.0 ± 0.2	4.0 ± 0.0^*^#	0.0 ± 0.0^*^#	5.5 ± 0.7^*^#	4.0 ± 0.0^*^#
*Fusarium avenaceum* (isolate 2)	0.0 ± 0.0	0.0 ± 0.0	3.5 ± 0.2^*^#	0.0 ± 0.0	2.0 ± 0.0^*^#	6.5 ± 0.2^*^#
*Nigrospora oryzae*	0.0 ± 0.0	0.0 ± 0.0	6.0 ± 0.4^*^#	0.0 ± 0.0	4.0 ± 0.5^*^#	3 ± 0.2^*^#
*Nigrospora sphaerica*	0.0 ± 0.0	0.0 ± 0.0	6.0 ± 0.6^*^#	0.0 ± 0.0	6.0 ± 0.2^*^#	3.5 ± 0.0^*^#
*Paraconiothyrium brasiliense*	0.0 ± 0.0	0.0 ± 0.0	5.0 ± 0.3^*^#	0.0 ± 0.0	4.0 ± 0.0^*^#	4.0 ± 0.0^*^#
*Penicillium afellutanum*	3.0 ± 0.2	3.0 ± 0.2	6.0 ± 0.6^*^#	0.0 ± 0.0^*^#	2.0 ± 0.5^*^#	5.0 ± 0.2^*^#
*Penicillium expansum*	2.0 ± 0.2	5.0 ± 0.2	3.0 ± 0.2^*^#	0.0 ± 0.0^*^#	4.0 ± 0.0^*^#	5.5 ± 0.5^*^#
*Penicillium olsonii*	1.5 ± 0.3	3.5 ± 0.3	1.0 ± 0.2^*^#	0.0 ± 0.0^*^#	1.5 ± 0.2 #	3.0 ± 0.6^*^#
*Rosellinia corticium*	2.0 ± 0.2	4.5 ± 0.3	7.0 ± 0.6^*^#	0.0 ± 0.0^*^#	3.0 ± 0.2^*^#	7.0 ± 0.2^*^#

*The asterisk indicates that the treatment means are significantly different from Nystatin at p ≤ 0.05. The number sign indicates that the treatment means are significantly different from Amphotericin B at p ≤ 0.05*.

### Molecular identification of candidate endophytic bacteria

16S rDNA and 23S rDNA sequencing were used for taxonomic identification of endophytic bacteria. BLAST searching against the Genbank database suggested that three of the candidate endophytes, 1D6, 3H9, and 4G4, most closely resemble *Paenibacillus* sp. while 4G12 resembles a *Citrobacter* sp. (Figure 2, Table S1). GenBank accession numbers for strains 1D6, 3H9, 4G12, and 4G4 using 16S rDNA are KM104866, KM104867, KM104868, and KM104869, respectively. GenBank accession numbers for strains 1D6, 3H9, 4G12, and 4G4 using 23S rDNA are KM387727, KM387728, KM387729, and KM387730, respectively. Phylogenetic tree data suggested that that the three *Paenibacillus* strains are *P. polymxa* (Figure 2B), and further suggested that strain 4G4 is different than 1D6 and 3H9.

### Scanning electron microscope (SEM) imaging of endophytes

**Figure 3:**
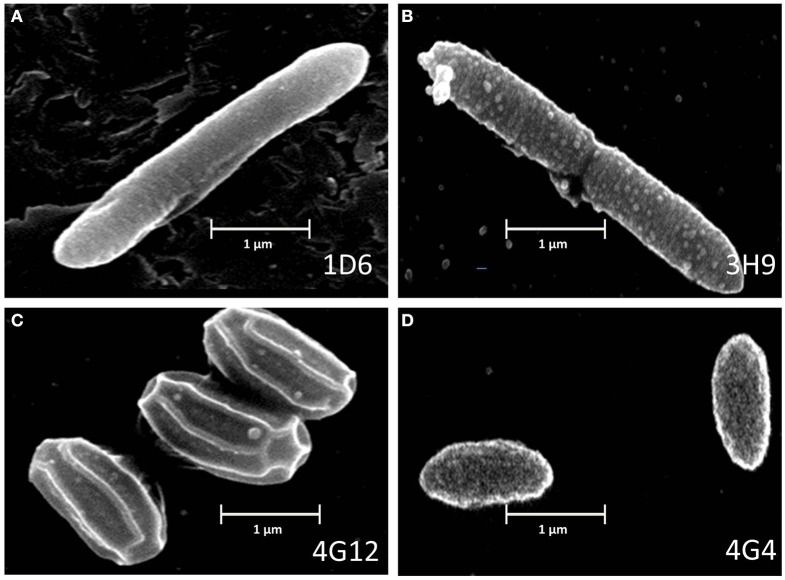
**Electron microscope images of the anti-fungal endophyte strains. (A–D)** correspond to strains 1D6, 3H9, 4G12, and 4G4, respectively.

Scanning electron microscopy was used to visualize the external appearance of the candidate endophytes (Figure 3). Of the three strains predicted to be *P. polymxa*, strain 1D6 was an elongated rod with an apparent smooth surface; strain 3H9 was an elongated rod with a rough surface; while strain 4G4 was a cylindrical rod with an apparently rough surface. The *Citrobacter*-predicted strain 4G12 had a rhomboidal hexagonal rod phenotype.

### *In vitro* interaction between each endophyte and *F. graminearum*

**Figure 4:**
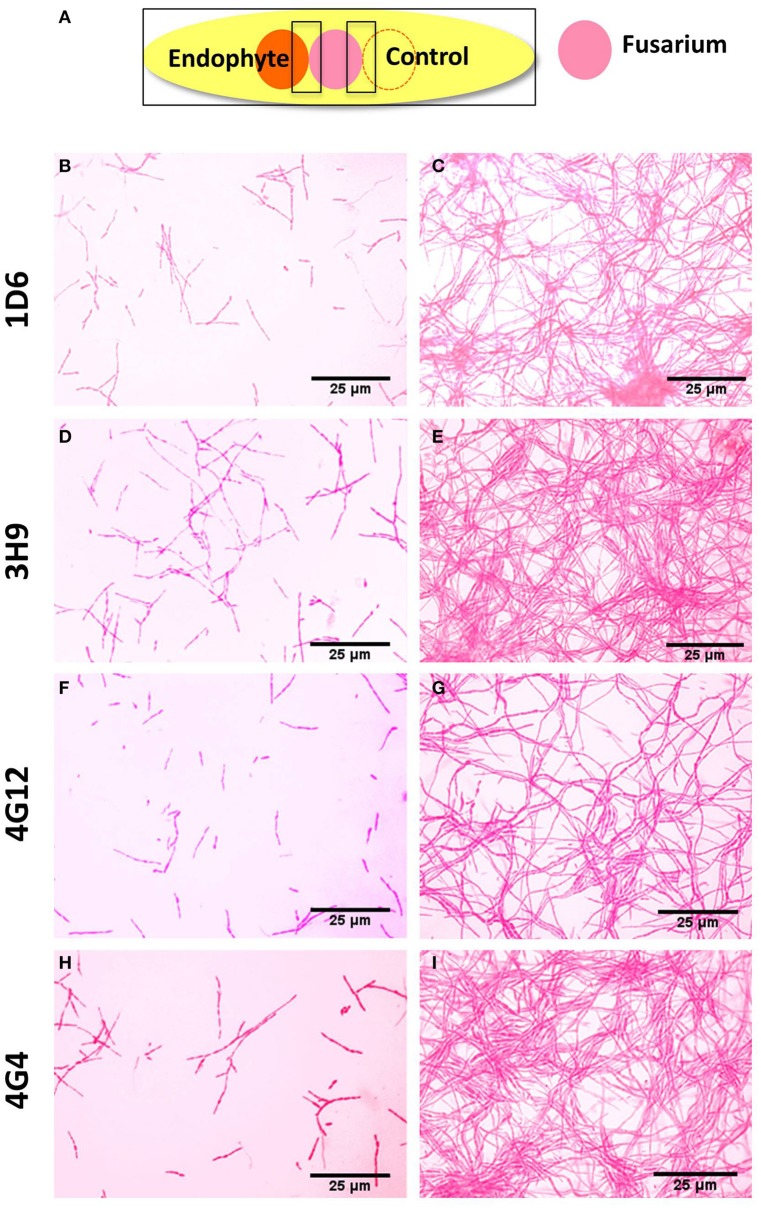
**Microscopic *in vitro* interactions between each anti-fungal endophyte and *F. graminearum*. (A)** Cartoon of the experimental methodology to examine microscopic *in vitro* interactions between *F. graminearum* (pink) and each endophyte (orange) or the buffer control (LB medium). The microscope slides were pre-coated with PDA and incubated for 24 h. *F. graminearum* hyphae were then stained with neutral red. Shown are representative microscope slide pictures (*n* = 3) of the interactions between *F. graminearum* and: **(B)** Strain 1D6 compared to **(C)** the buffer control; **(D)** Strain 3H9 compared to **(E)** the buffer control; **(F)** Strain 4G12 compared to **(G)** the buffer control; **(H)** Strain 4G4 compared to **(I)** the buffer control.

**Figure 5:**
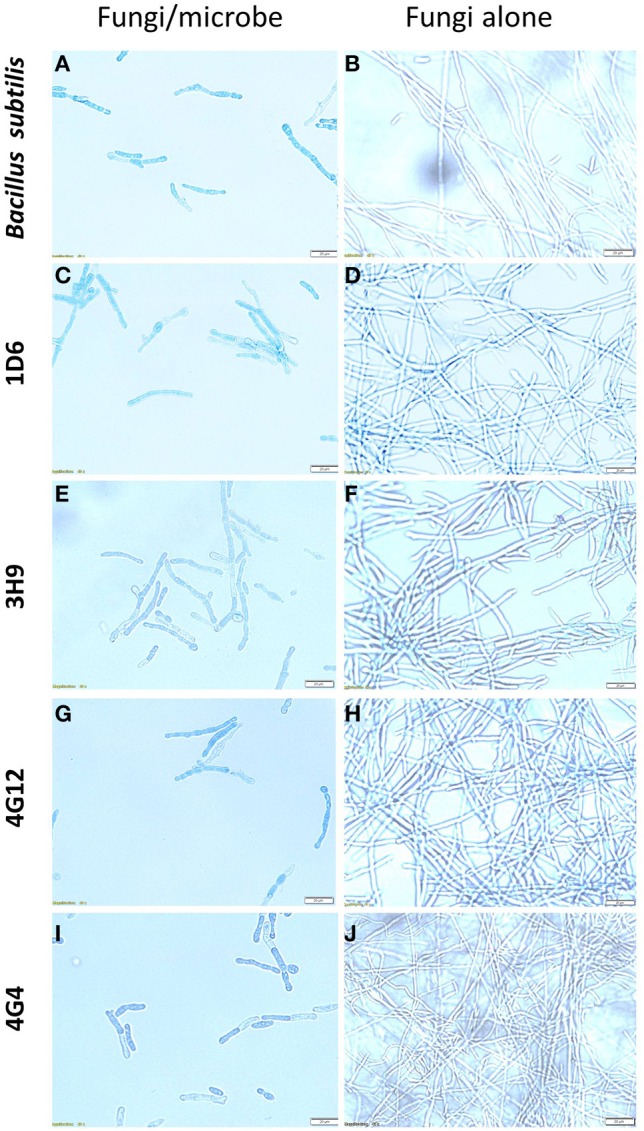
**The effects of the candidate endophytes on *F. graminearum in vitro* using the vitality stain, Evans blue**. Shown are representative microscope slide pictures (*n* = 3) of the interactions of *F. graminearum* with: **(A)** the commercial biological control agent, *Bacillus subtilis* (100 mg/10 ml) compared to **(B)** the buffer control; **(C)** Strain 1D6 compared to **(D)** the buffer control; **(E)** strain 3H9 compared to **(F)** the buffer control; **(G)** Strain 4G12 compared to **(H)** the buffer control; **(I)** Strain 4G4 compared to **(J)** the buffer control.

To better understand the anti-fungal mode of action of the candidate endophytes, the *in vitro* interactions between *F. graminearum* and each endophyte were visualized following their co-incubation on a microscope slide and subsequent staining with the vitality stains, neutral red and Evans blue. All the four endophytes caused apparent dramatic breakage of *F. graminearum* hyphae when compared to the control zone on the other side of the microscope slide (that was exposed to only LB media) (Figure 4). Upon staining with Evans blue (which stains dead cells in blue), fungal hyphae in contact with the commercial biological control or each of the four endophytes stained blue (Figures 5A,C,E,G,I) compared to the buffer controls (Figures 5B,D,F,H,J), suggesting that hyphae in contact with each bacterial endophyte died. Combined, these results suggest that all four endophytes have fungicidal activity.

### Candidate fungicide mechanism of action

**Figure 6:**
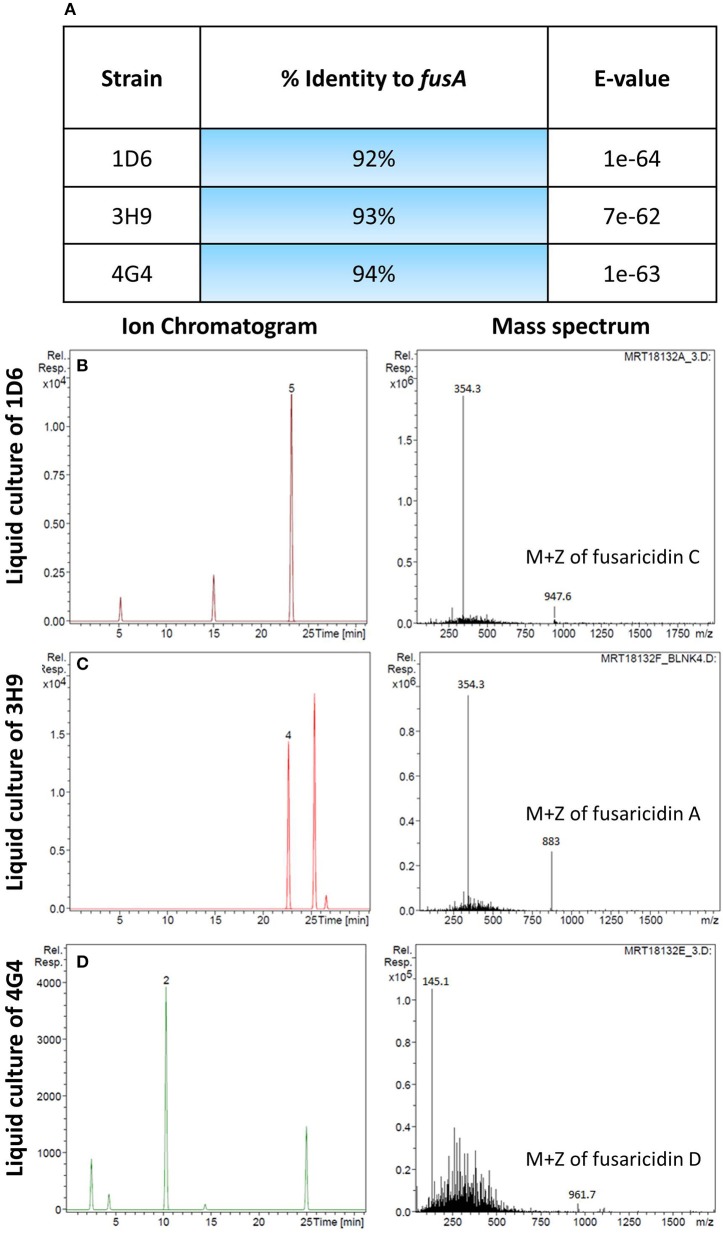
**Molecular and biochemical detection of the candidate anti-fungal compound, fusaricidin, in *Paenibacillus* strains. (A)** Details of *fusA* gene orthologs isolated from the candidate *Paenibacillus* endophytes by PCR amplification. **(B–D)** Combined ion chromatogram/mass spectrum for candidate fusaricidin derivatives detected in the cultures of the *Penibacillus* endophytes as indicated.

A candidate gene approach was undertaken to help understand the fungicide mode of action of the endophytes. *Paenibacillus* are well known to produce fusaricidin compounds that combat various fungal pathogens including *F. graminearum*; the compound is in fact named after *Fusarium* (Kajimura and Kaneda, 1996, 1997; Beatty and Jensen, 2002; Choi et al., 2008). To detect the presence of fusaricidin biosynthetic genes in the three predicted *Paenibacillus* strains, PCR primers were designed based on the fusaricidin synthase gene sequence (GenBank accession # EU184010). Each genome amplified a single band that was sequenced; the results revealed that all three of the *Paenibacillus* endophyte genomes encode a putative *fusA* ortholog, with DNA sequence identities ranging from 92 to 94% (Figure 6A, Table S2). GenBank accession numbers for *fusA* sequences amplified from strains 1D6, 3H9, 4G4 were KT343965, KT343966, and KT343967, respectively. *FusA* is a non-ribosomal peptide synthetase with relaxed substrate specificity that can incorporate different amino acids, resulting in different fusaricidin derivatives (Han et al., 2012). To confirm that the *fusA* orthologs were expressed by the endophytes, and to identify the specific fusaricidin derivatives produced, LC/MS was employed. Peaks with M + Z similar to fusaricidin C (947.6), fusaricidin B (883), and fusaricidin D (961.7) were detected in the liquid cultures of strains 1D6, 3H9, and 4G4, respectively (Figures 6B–D) (Kajimura and Kaneda, 1996, 1997; Beatty and Jensen, 2002). Combined, these results demonstrate that the well-known anti-*Fusarium* compound fusaricidin is encoded and expressed by each of the three *Paenibacillus* endophytes.

### Suppression of gibberella ear rot (GER) *in planta*

**Figure 7:**
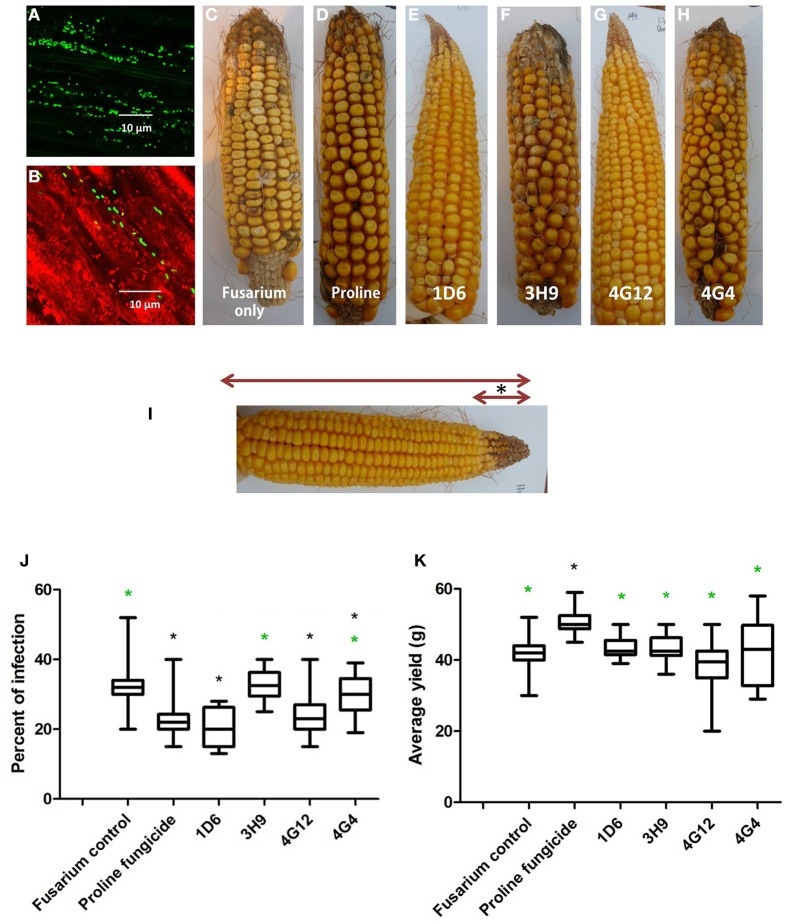
**Greenhouse trial 1 to test for the ability of the candidate endophytes to suppress Gibberella Ear Rot (GER) in a modern hybrid. (A, B)** GFP-tagged endophyte strain 4G12 visualized inside maize roots, in the **(A)** absence or **(B)** presence of propidium iodide that outlines the cell with red color. **(C–H)** Representative ears from each treatment. **(I)** Picture of an ear to illustrate the methodology of scoring disease severity: The fungal pathogen was introduced to the tip of the ear, indicated by the asterisk. Therefore, the disease was scored as the ratio of the length of the diseased ear tip portion relative to total ear length, multiplied by 100 to give a percentage. **(J, K)** Quantification of the effect of different treatments on GER suppression, as: **(J)** percent ear infection, and **(K)** average grain yield per plant. For both measurements, *n* = 20 per treatment (*n* = 10 for both controls). The whiskers indicate the range of data points. The black asterisk indicates that the treatment means were significantly different from the *Fusarium* only treatment at *p* ≤ 0.05. The green asterisk indicates that the treatment means were significantly different from prothioconazole fungicide (Proline) treatment at *p* ≤ 0.05.

**Figure 8:**
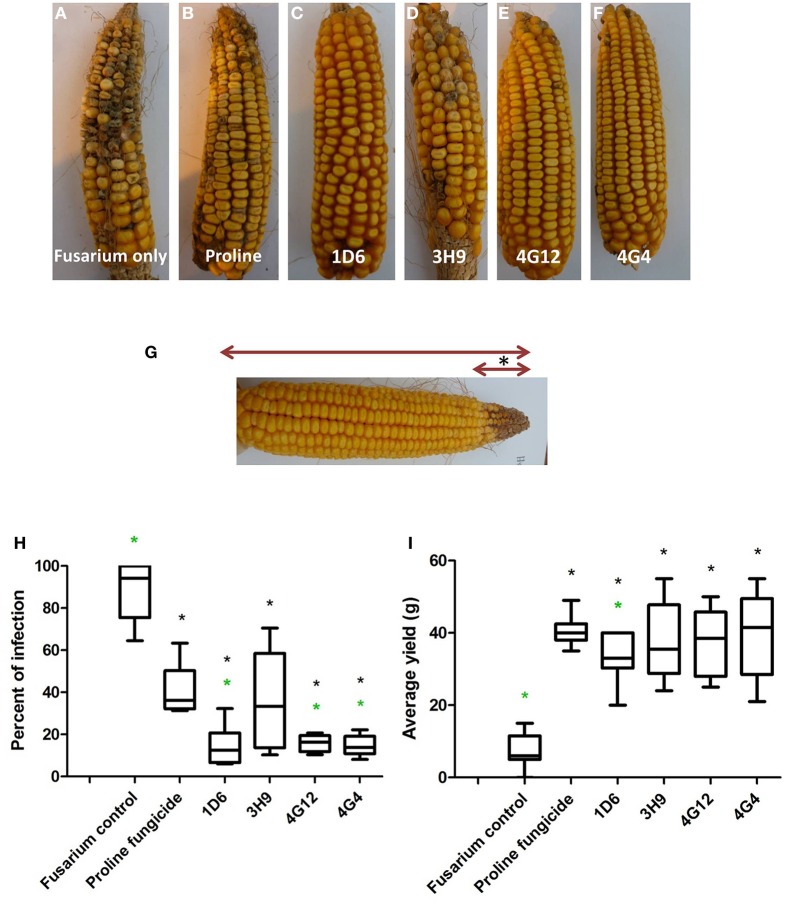
**Greenhouse trial 2 to test for the ability of the candidate endophytes to suppress Gibberella Ear Rot in a modern hybrid**. **(A–F)** Representative ears from each treatment. **(G)** Picture of an ear to illustrate the methodology of scoring disease severity: the fungal pathogen was introduced to the tip of the ear, indicated by the asterisk. Therefore, the disease was scored as the ratio of the length of the diseased ear tip portion relative to total ear length, multiplied by 100 to give a percentage. **(H, I)** Quantification of the effect of different treatments on GER suppression, as **(H)** percent ear infection, and **(I)** average grain yield per plant. For both measurements, *n* = 20 per treatment (*n* = 10 for both controls). The whiskers indicate the range of data points. The black asterisk indicates that the treatment means were significantly different from the *Fusarium* only treatment at *p* ≤ 0.05. The green asterisk indicates that the treatment means were significantly different from prothioconazole fungicide (Proline) treatment at *p* ≤ 0.05.

Greenhouse experiments were undertaken to determine if the endophytes could suppress Gibberella ear rot (GER) *in planta* using a modern maize hybrid, P35F40, which is susceptible to this disease. To confirm that the candidate bacterial strains originally isolated from the two evolutionarily distant maize genotypes (*Z. diploperennis* and Parviglumis) could colonize the internal tissues of this modern hybrid, thus behaving as endophytes, GFP tagging was conducted. Attempts were made to GFP tag all endophytes, but unfortunately, only strain 4G12 from ancestral Parviglumis, was successfully tagged. GFP-tagged 4G12 was visualized by scanning confocal microscopy and shown to colonize maize roots (Figures 7A,B), confirming its behavior as an endophyte in the modern maize relative. All four endophytes were then tested for their ability to suppress GER under greenhouse conditions in two independent trials (Figures 7, 8). The main entrance routes for *F. graminearum* in maize are exposed silks where the ascospores can germinate and grow toward the developing ear (Sutton, 1982; Kebebe et al., 2015). Therefore, the disease severity was scored as the length of diseased area, measured from the ear tip where *Fusarium* spores were introduced, relative to the total length of the ear (Figure 7I). Kernel yields were also quantified:

#### First greenhouse trial (summer 2012)

Representative pictures of treated ears are shown (Figures 7C–H). Treatment with three of the four endophytes caused significant reductions (*P* ≤ 0.05) in GER disease severity ranging from 12 to 38%: strain 1D6 resulted in the greatest disease suppression followed by strain 4G12 and then strain 4G4, while the effect of strain 3H9 on GER suppression was statistically insignificant when compared to the *Fusarium* treatment only, at *P* < 0.05 (Figure 7J, Table 2). None of the endophyte treatments caused a significant change in grain yield, at *P* ≤ 0.05 (Figure 7K, Table 2).

**Table 2:** **Suppression of Gibberella Ear Rot by the candidate endophytes in two replicate greenhouse trials**.

**Treatment**	% infection (mean ± SEM)^*^	**% disease reduction relative to *Fusarium* only treatment**	Average yield per plant (g)^*^	**% yield increase relative to *Fusarium* only treatment**
**GREENHOUSE TRIAL 1**				
*Fusarium* only	33.7 ± 2.3 a	0.0	41.7 ± 1.3 a	0.0
Proline fungicide	23.1 ± 2.0 b	31.5	50.9 ± 1.2 b	22
1D6	20.9 ± 1.7 b	38	43.5 ± 1.2 a	4.3
3H9	32.4 ± 1.4 a	3.9	43.1 ± 1.3 a	3.4
4G12	24.1 ± 2.1 b	28.5	38.3 ± 2.5 a	−8.2
4G4	29.7 ± 1.7 d	11.9	42.5 ± 2.9 a	1.9
**GREENHOUSE TRIAL 2**				
*Fusarium* only	88.5 ± 3.8 a	0.0	7.7 ± 1.2 a	0.0
Proline fungicide	41.4 ± 4.5 b	53.2	40.8 ± 1.2 b	429.9
1D6	14.6 ± 1.9 c	83.5	33.5 ± 2.1 c	335
3H9	36.1 ± 6.2 b	59.2	37.5 ± 3.3 b	387
4G12	16 ± 1.1 c	81.9	37.4 ± 3.2 b	385.7
4G4	14.6 ± 1.3 c	83.5	39.8 ± 3.0 b	416.9

* *Letters that are different from one another indicate that their means are statistically different (P ≤ 0.05)*.

#### Second greenhouse trial (summer 2013)

In the second trial, the disease pressure was increased by raising the humidity. Representative pictures of treated ears are shown (Figures 8A–F). Treatments with all four of the endophytes caused significant reductions (*P* ≤ 0.05) in GER disease severity ranging from 59 to 84%: strains 1D6, 4G12, and 4G4 resulted in the statistically greatest disease suppression, while again the effect of strain 3H9 on GER suppression was the lowest, but this time statistically significant compared to the *Fusarium* treatment only, at *P* < 0.05 (Figure 8H, Table 3). All of the endophyte treatments caused dramatic 3-4-fold increases in grain yield compared to the *Fusarium* treatment only (Figure 8I, Table 2).

**Table 3:** **Reduction of DON mycotoxin accumulation during storage following treatment with the candidate endophytes**.

**Treatment**	DON content (ppm) (mean ± SEM)^*^	% of DON reduction relative to *Fusarium* only treatment^*^
**GREENHOUSE TRIAL 1**		
*Fusarium* only	3.4 ± 0.4 a	0.0
Proline fungicide	0.7 ± 0.4 b	79.4
1D6	0.1 ± 0.0 c	97
3H9	1.0 ± 0.8 d	70.6
4G12	0.1 ± 0.0 c	97
4G4	0.1 ± 0.0 c	97
**GREENHOUSE TRIAL 2**		
*Fusarium* only	3.5 ± 0.3 a	0.0
Proline fungicide	0.1 ± 0.0 b	97.1
1D6	0.1 ± 0.0 b	97.1
3H9	0.1 ± 0.0 b	97.1
4G12	0.1 ± 0.0 b	97.1
4G4	0.2 ± 0.1 b	94.3

* *Letters that are different from one another indicate that their means are statistically different (P ≤ 0.05)*.

### Effect of the endophyte treatments on DON contamination

**Figure 9:**
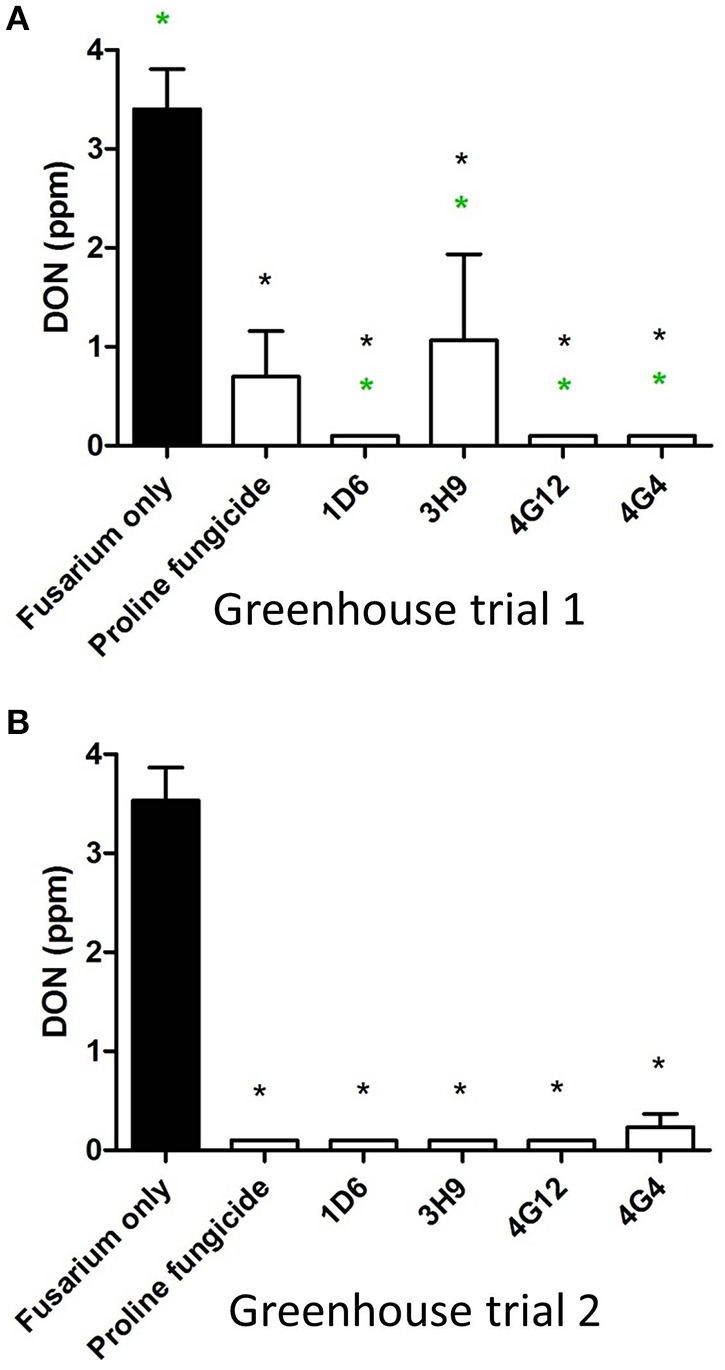
**Test for the ability of the candidate endophytes to reduce DON mycotoxin accumulation in maize grain during storage**. DON measurements after storage of maize grain from: **(A)** greenhouse trial 1 (summer 2012), and **(B)** greenhouse trial 2 (summer 2013). For both trials, *n* = 3 pools of seeds. The black asterisk indicates that the treatment means were significantly different from the *Fusarium* only treatment at *p* ≤ 0.05. The green asterisk indicates that the treatment means were significantly different from the prothioconazole fungicide (Proline) treatment at *p* ≤ 0.05.

In order to quantify DON levels in maize seeds, ELISA-based testing was conducted. Immediately after harvest, only traces of DON were detected in plants treated with *Fusarium* only (approximately 0.1 ppm) while all other treatments did not show any detectable levels of DON (data not shown). Seeds were stored at room temperature inside closed envelopes for one year, then the samples were analyzed again for DON content. Consistently in both trials, all four endophyte treatments caused dramatic reductions in DON accumulation during storage, with DON levels declining from approximately 3.5 ppm to 0.1–1.0 ppm (Figure 9, Table 3). The majority of the endophyte treatments resulted in a DON content of only 0.1 ppm, equivalent to a 97% reduction compared to the *Fusarium*-only control.

### DON detoxification

In order to test the ability of the candidate endophytes to directly detoxify DON into epi-DON *in vitro*, a standard HPLC based method was used. However, the results revealed that none of the candidate endophytes could directly detoxify DON into epi-DON (Figure S1).

## Discussion

We hypothesized that the wild relatives of modern crops, which grow without fungicides, may host beneficial endophytes that help their hosts to naturally combat fungal pathogens including *F. graminearum*. In this study we found support for this hypothesis. *In vitro* screening of 215 maize bacterial endophytes (Figures 1A,B) identified four candidate endophytes that could inhibit the growth of *F. graminearum* and its DON mycotoxin to within the acceptable levels (Figure 9). Despite the screen containing 116 endophytes from non-wild maize genotypes (45 from modern maize and 71 from traditional landraces, totaling 54% of the library), the three most potent endophytes were isolated from their wild counterparts (representing 46% of the endophytes screened) (Figures 1, 7, 8). Specifically, anti-*Fusarium* endophyte strain 1D6 was originally isolated from *Z. diploperennis*, and strains 4G12 and 4G4 were isolated from Parviglumis (Figure 2A). The remaining candidate endophyte (strain 3H9) was isolated from a modern maize variety (*Z. mays* ssp. *mays*, Pioneer 3751 hybrid). The suggested mode of action of all four endophytes was fungicidal, not fungistatic (Figures 4, 5). Plants treated with these endophytes showed a remarkable reduction in DON contamination during storage (Figure 9) which might be attributed to an initial reduction in *F. graminearum* inoculum, as none of the endophytes were able to directly inactivate DON *in vitro* (Figure S1). The permitted level of DON mycotoxin contamination in maize grain is 2 ppm in food and 5 ppm in animal feed (Jelinek et al., 1988). However, the dietary value permitted for swine feed in Canada and the USA is only 1 ppm (Schaafsma et al., 2009). Except for strain 3H9, the endophytes consistently reduced DON to within the 1 ppm level during storage.

### Host environmental history

Interestingly, the anti-*Fusarium* endophytes isolated from the wild teosintes (1D6, 4G12, and 4G4) showed an exceptionally broad spectrum of anti-fungal activities (Table 1). Parviglumis teosinte appears to have been adapted for thousands of years in the seasonal tropical forest region of the Central Balsas Valley of southwestern Mexico (Piperno et al., 2009). *Zea diploperennis* originated from the Sierra de Manantlan region of Jalisco, in Southern Mexico, which has both dry and wet climates but with very high levels of total rainfall (1700 mm) (Iltis and Doebley, 1980; Sánchez−Velásquez et al., 2002). Perhaps the broad spectrum activity of the endophytes from these *Zea* genotypes is the result of co-evolutionary selection by their host plants for endophytes that could combat fungal pathogens which are especially problematic in regions of high humidity. In contrast, the candidate endophyte (strain 3H9) isolated from the modern hybrid, which was bred under temperate conditions, showed the weakest anti-*Fusarium* activities (Figures 1D, 7J, 8H, 9A) as well as the narrowest target spectrum of anti-fungal activity (Table 1).

Unfortunately, there has been no systematic comparison of fungal resistance in teosintes vs. modern maize, though one *Fusarium* species (*Gibberella fujikori*) has been reported to infect both (Lange et al., 2014). In a survey concerning the incidence of *Fusarium* species in maize seeds worldwide, *Fusarium* species were the most abundant fungi detected (39–62%) with the most frequent species being *F. moniliforme* (MacDonald and Chapman, 1997). *Fusarium* species were reported in 10% of wild teosinte seeds collected from Mexico, Nicaragua and Guatemala, with *F. moniliforme* and *F. subglutinans* reported to be the most abundant (Desjardins et al., 2000). However, the authors noted that whereas *Fusarium* species were detected in 100% of modern maize seeds (*Z. mays* spp. *mays*), their incidence in teosinte seeds was only 4% when grown under the same conditions, and in general, at least anecdotally, teosintes appeared to have much lower rates of *Fusarium* infection than modern maize in Mexico (Desjardins et al., 2000). It may be that there has been three-way co-evolutionary selection within the teosintes between the host plant, its endophytes and *Fusarium* species.

### Host life strategy

This study involved *Zea* genotypes which encompassed three critical life strategy transitions: (1) the evolutionary transition from wild perennial to annual growth habit; (2) the agricultural transition from wild to domesticated primitive plant; and (3) the transition from a domesticated primitive plant to modern cultivars (Rosenthal and Dirzo, 1997; Dávila-Flores et al., 2013). As noted above, the three consistently robust anti-fungal endophytes were isolated from *Z. diploperennis*, which is a wild perennial teosinte, and from Parviglumis, which is a wild annual teosinte. These two *Zea* genotypes were previously shown to be more resistant to insects compared to domesticated modern maize (an annual), with *Z. diploperennis* showing more resistance than Parviglumis (Rosenthal and Dirzo, 1997; Dávila-Flores et al., 2013). These results are consistent with other reports that domestication reduces resistance to insects (Lange et al., 2014) and herbivores (Chen et al., 2015). To explain these results, Rosenthal and Dirzo (1997) suggested the resource allocation hypothesis in which metabolic resources are diverted away from plant defense as a result of selection for faster plant growth rates (associated with annualism) and higher grain yields (associated with domestication and breeding). This hypothesis is supported by results from other crops (Benrey et al., 1998; Gols et al., 2008). Given the results of this study, it is interesting to pose a parallel hypothesis: as plants diverted precursors for defense compounds to enable faster plant growth and higher grain yield, they may have also prevented their endophytes from producing defense compounds, thus reducing the reasons to support such endophytes. Modern breeding under conditions of fungicide inputs may have also caused crops to no longer devote metabolic resources to support endophytes with redundant pesticide function. Alternatively, it may be that selection by humans against plant-derived toxins during domestication and breeding may have also involved selection against anti-pathogen endophytes with indiscriminate toxicity, by altering plant loci that promote the colonization of specific endophytes. These three hypotheses (host-endophyte resource allocation hypothesis, pesticide-endophyte-redundancy hypothesis, endophyte-toxin-selection hypothesis) require further investigation. As this study involved only two modern maize genotypes (B73, Pioneer 3751) focusing on only a single pathogen, future studies should involve a more balanced number of plant genotypes across the evolutionary spectrum.

### *Paenibacillus polymyxa* strains span the evolutionary transitions of *zea*

In this study, despite testing 215 diverse bacterial endophyte strains, three out of four candidate endophytes with anti-*Fusarium* activity were predicted to be *Paenibacillus polymyxa* (strains 1D6, 3H9, and 4G4) with a fourth strain identified as a *Citrobacter* sp. (strain 4G12) (Figure 2, Table S1). The three *P. polymyxa* appear to be distinct, when all the data are taken into account [16S rDNA phylogenetic tree data (Figure 2B), *fusA* gene sequence information (Figure 6A, Table S2), biochemical profiles (Figure 6), and anti-*Fusarium* results (e.g., Figure 7)]. Bacterial endophytes previously isolated from different maize varieties belong to diverse genotypes including *Paenibacillus* and *Citrobacter*, but also *Bacillus, Clostridium, Enterobacter, Pantoea, Methylobacteria, Pseudomonas, Burkholderia, Erwinia*, and *Microbacterium* (Johnston-Monje and Raizada, 2011; Cotta et al., 2014; Johnston-Monje et al., 2014).

As already noted, the putative *P. polymyxa* endophytes appear to span hundreds of thousands of years of evolutionary transitions of *Zea* (Matsuoka et al., 2002), since they were present in a wild Mexican perennial (*Z. diploperennis*), a wild Mexican annual (Parviglumis) and a modern temperate hybrid (Pioneer 3751) (Table 2). Therefore, these anti-*Fusarium Paenibacilli* cross host boundaries of evolution, domestication, migration and breeding, suggesting a tight conserved host-endophyte relationship. Consistent with this observation, *P. polymyxa* was previously reported as a ubiquitous, conserved endophyte across diverse *Zea* genotypes including wild perennial and annual teosintes, traditional farmer landraces and modern genotypes (Johnston-Monje and Raizada, 2011). *P. polymyxa* is well known as a good plant colonizer, which is in part due to its robust ability to form biofilms (Timmusk et al., 2005; Haggag and Timmusk, 2008). Furthermore, consistent with our results, *P. polymyxa* was previously reported to antagonize numerous plant pathogens (Timmusk et al., 2009; Xu and Kim, 2014) including *F. graminearum* (He et al., 2009). *P. polymyxa* was further shown to decrease DON production under greenhouse conditions (He et al., 2009), consistent with the results of this study.

Previous reports have shown that the anti-fungal mechanism of action of *Paenibacillus* sp. involves production of potent antifungal compounds including polymyxins, fusaricidins, colistins, volatiles, and lytic enzymes (He et al., 2007; Raza et al., 2008, 2009, 2015; Naghmouchi et al., 2012). In particular, *Paenibacillus* is well-known to combat *F. graminearum* by employing fusaricidin, a compound named after *Fusarium* as noted earlier (Kajimura and Kaneda, 1996, 1997; Beatty and Jensen, 2002; Choi et al., 2008). Consistent with the literature, our results show that the three *P. polymyxa* strains characterized in this study produce fusaricidins (Figure 6, Table S2). However, we do not exclude other compounds as the genus *Paenibacillus* is well-known for its ability to produce an arsenal of antimicrobial compounds including polyketides and non-ribosomal peptides. Fusaricidin production, together with the *in vitro* microscopic interaction data (Figures 4, 5, 6), suggests a fungicidal mechanism of action. *P. polymyxa* has also been shown to induce systemic host resistance (Mei et al., 2014). *P. polymyxa* was also shown to alter plant metabolism by enhancing the production of flavonoids such as apigenin-7-O-glucoside (Schmidt et al., 2014) or reducing cinnamic acid in root exudates. These compounds suppress the development of pathogen conidia, thereby significantly reducing their ability to colonize plants (Ling et al., 2011). It will be useful to investigate if the predicted *Paenibacilli* endophytes from this study also employ these modes of action. It will also be useful to test whether these strains are resistant to fusaric acid, an antibiotic produced by *Fusarium*; resistance to this compound has been shown to be essential for effective biological control against *Fusarium* species (Bacon and Hinton, 2011).

### The emerging importance of *citrobacter* sp.

As already noted, the anti-*Fusarium* endophyte strain 4G12 is predicted to be a *Citrobacter* species (Figure 2). *Citrobacter* species were previously reported as endophytes of an ancient Mexican landrace (Nal Tel) and teosinte (e.g., *Z. nicaraguensis*) (Johnston-Monje and Raizada, 2011), in addition to other plants including Brazilian sugarcane (Magnani et al., 2013) and the legume tree, *Conzattia multixora* which is exclusively found in Guatemala and Mexico (Wang et al., 2006). *Citrobacter* species were claimed to moderately control some fungal plant pathogens such as *Monilinia fructicola*, the causal agent of brown rot in stone fruits (Janisiewicz et al., 2013). However, to the best of our knowledge, *Citrobacter* was not previously reported to effectively control *Fusarium* species, suggesting that this genus may have a wider spectrum of anti-fungal activity than previously thought (Table 1).

## Conclusions

This study has identified candidate endophytes that could suppress the serious, toxigenic fungal pathogen *F. graminearum* in maize. The endophytes reduced DON mycotoxin concentrations during storage to levels significantly below acceptable safety thresholds, a promising result that requires field level validation for further practical applications. The most potent of the candidate strains were derived from wild teosinte genotypes including the ancestor of modern maize. Further bioprospecting of wild relatives of modern crops for beneficial microbes may open a promising avenue for biocontrol against the most devastating diseases afflicting modern agriculture. Teosintes are under threat from deforestation, urbanization, and cattle, and it is hoped that this study will assist in efforts to conserve these wild species. These results, combined with the literature, have led us to several host-endophyte hypotheses with respect to plant defense that require further investigation.

## Author contributions

WM conducted all experiments and analyzed all data, but was assisted by CS for the greenhouse trials, and VL who performed the DON quantification experiments. TZ designed the DON detoxification experiment. WM wrote the manuscript, and MR edited the manuscript. All authors read and approved the manuscript.

### Conflict of interest statement

The authors declare that the research was conducted in the absence of any commercial or financial relationships that could be construed as a potential conflict of interest.

## Supplementary material

Figure S1 **Test for the ability of the candidate endophytes to detoxify DON *in vitro***. Shown are representative HPLC chromatograms showing that none of the endophyte treatments caused DON detoxification. Shown are treatments with: **(A)** the buffer control (LB media), **(B)** endophyte 1D6, **(C)** endophyte 3H9, **(D)** endophyte 4G12, and **(E)** endophyte 4G4.

Table S1 **Taxonomic classification of maize bacterial endophytes based on 16S rDNA and 23S rDNA sequences and BLAST analysis (corresponding to Table 1) (Excel file)**.

Table S2 **Sequences of *fusA* orthologs from *Paenibacillus* endophytic species identified in this study (corresponds to Figure 4A) (Excel file)**.

Table S3 **Origin of the endophytes screened in this study including the maize genotypes and tissues from which they were isolated, and the best BLAST matches to the GenBank database (Excel file)**.
