# Supplementary figures and images for: Bacterial endophytes from wild maize suppress Fusarium graminearum in modern maize and inhibit mycotoxin accumulation
Source: Front Plant Sci. 2015 Oct 6;6:805. doi: 10.3389/fpls.2015.00805 (PMC4593954; doi:10.3389/fpls.2015.00805)

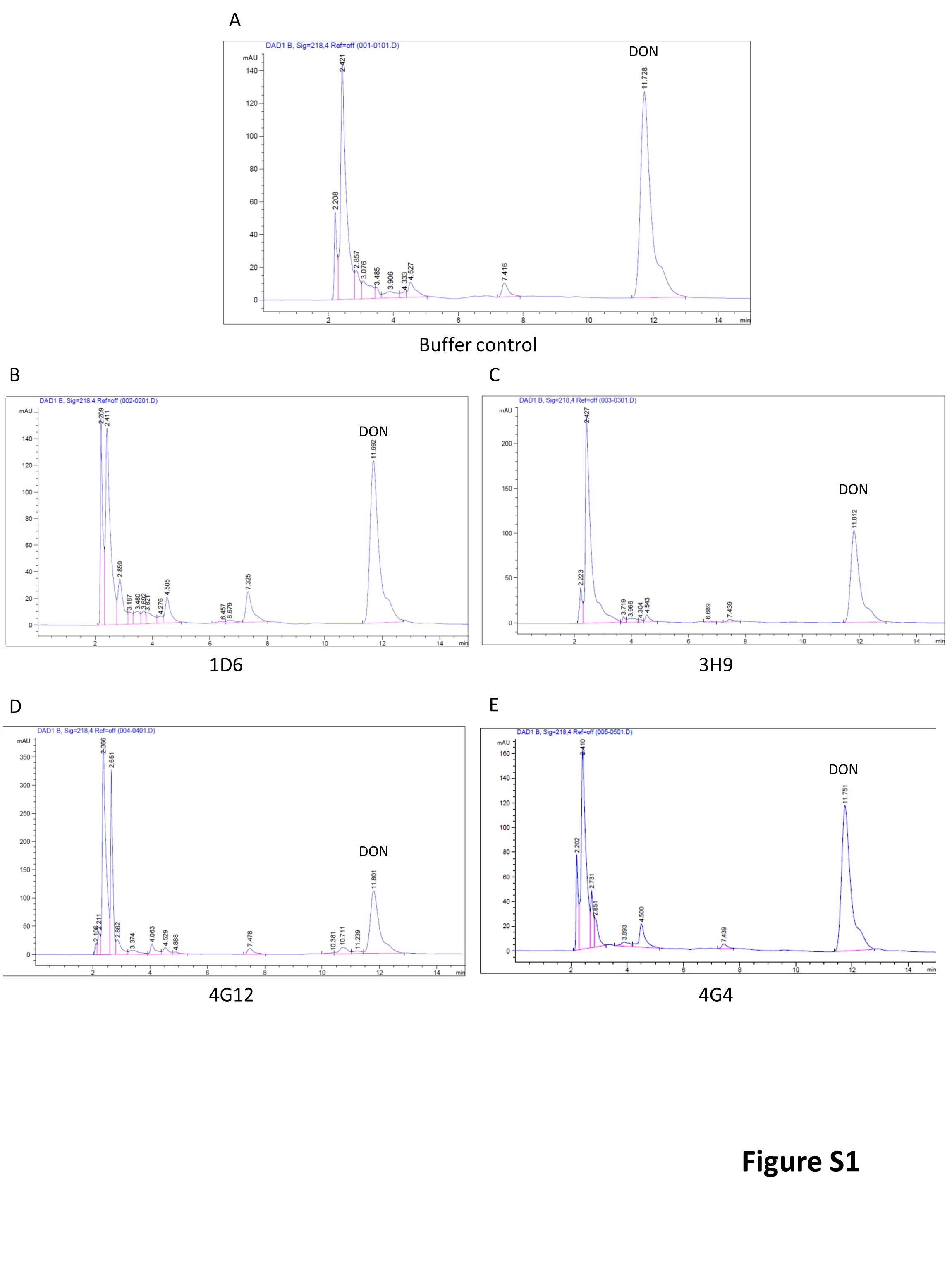

Supplement: Figure S1 — Test for the ability of the candidate endophytes to detoxify DON in vitro. Shown are representative HPLC chromatograms showing that none of the endophyte treatments caused DON detoxification. Shown are treatments with: (A) the buffer control (LB media), (B) endophyte 1D6, (C) endophyte 3H9, (D) endophyte 4G12, and (E) endophyte 4G4. [file Image1.JPEG]
